# Spatial typologies in psychiatric facilities

**DOI:** 10.1192/j.eurpsy.2023.169

**Published:** 2023-07-19

**Authors:** J. Kirch

**Affiliations:** a|sh architekten, Ludwigshafen, Germany

## Abstract

**Abstract:**

A psychiatric facility represents a temporary home for its patients. Multiple studies have provided ample evidence that the built environment has the potential to support patients in their recovery process, in part by offering a homely surrounding. If their environment also succeeds in creating a therapeutic milieu in which the patients’ needs for protection, security, privacy, and orientation are met, the contribution of these surroundings can be even more significant. For example, clear, comprehensible building structures help patients to find their way around the new environment and further provide a feeling of security. To fulfill this goal, planners should pay special attention to the access zones and semi-public spaces in these types of buildings, as psychiatric patients often spend a lot of time there. Corridors should provide high spatial quality with daylight areas and places to sit down.

Based on an analysis of more than 30 psychiatric facilities in Germany, three spatial typologies were identified within which the factors listed above have been explored.

Firstly, the “Pavilion type”: square or slightly rectangular pavilion structures, generally with courtyards enclosed on four sides and multiple additions. This typology is found very often, especially on new build sites. The Pavilion type allows a useful combination of room functions and good lighting of all spaces. Secondly, the “L- or T-shaped type”: Linked L- or T-shaped, often appearing as comb-like buildings. These structures are particularly successful in integrating with the surrounding landscape. Thirdly, the “Block type”: Closed, block-like single-floor and two-floor typologies of different lengths. However, these building structures are increasingly rare as they often appear out of human scale and result in long, monotonous corridors.

In building design it is crucial to consider the triad of “architecture/interior design/ and landscape design” and to emphasize the specifics of the site. Each of these typologies offer different opportunities to achieve this goal; yet, only when a unique atmosphere is created – one in which everyone feels accepted and is seen as an individual – can patients, staff and visitors feel the comfort and support of a successful homely environment.

**Disclosure of Interest:**

None Declared

